# Single-Phase PacBio *De Novo* Assembly of the Genome of the Chytrid Fungus *Batrachochytrium dendrobatidis,* a Pathogen of Amphibia

**DOI:** 10.1128/MRA.01348-18

**Published:** 2018-11-29

**Authors:** Nicholas Sumpter, Margi Butler, Russell Poulter

**Affiliations:** a Department of Biochemistry, University of Otago, Dunedin, New Zealand; University of Maryland School of Medicine

## Abstract

Here, we present an updated genome assembly of the diploid chytrid fungus Batrachochytrium dendrobatidis strain RTP6. This strain is part of the global panzootic lineage (*Bd*GPL) and was isolated in Dunedin, New Zealand. The assembly was generated using PacBio long-read and Illumina short-read data, allowing for the accurate phasing of heterozygosities.

## Abstract (precis)

Here, we present an updated genome assembly of the diploid chytrid fungus Batrachochytrium dendrobatidis strain RTP6. This strain is part of the global panzootic lineage (*Bd*GPL) and was isolated in Dunedin, New Zealand.

## ANNOUNCEMENT

Batrachochytrium dendrobatidis is a diploid chytrid fungus that causes amphibian chytridiomycosis, a skin disease resulting in the decline and extinction of amphibian species globally (1–3). Previous genome assemblies of Batrachochytrium dendrobatidis global panzootic lineage (*Bd*GPL) isolates (4) used Sanger sequencing (GenBank assembly accession numbers GCA_000149865 and GCF_000203795). Heterozygous sites in these assemblies were randomly assigned, resulting in “pseudohaploid” genome assemblies representing partial chromosomes.

This study describes a single-phase genome assembly of B. dendrobatidis strain RTP6 that used PacBio long-read technology (5). RTP6 was isolated from a Litoria ewingii tadpole (Dunedin, New Zealand) by swabbing its keratinized mouthparts and was cultured on modified tryptone gelatin hydrosylate lactose (mTGhL) agar (6). DNA isolation was performed using a modified chloroform extraction protocol with 10% SDS as a surfactant and avoiding mechanical shearing to preserve DNA length. SMRTbell library preparation was performed by Macrogen, Inc. (files.pacb.com/Training/IntroductiontoSMRTbellTemplatePreparation/story_content/external_files/Introduction%20to%20SMRTbell%E2%84%A2%20Template%20Preparation.pdf). Macrogen also performed the PacBio RS II sequencing and read quality control and processing using standard procedures (pacb.com/training/PostRunQCAnalysis/story_content/external_files/Post%20Run%20QC%20Analysis.pdf). A total of 161,546 subreads (average length, 7,516 bp) were obtained and then *de novo* assembled using Canu version 1.5 (7), producing 106 contigs with a total length of 24.656 Mb (*N*_50_, 653 kb). Subsequent analyses were performed using Geneious software version 10.2.3. A number of methods were used to gain evidence for manual fusion or extension of contigs. These included comparison to previous assemblies, remapping of strain RTP6 PacBio long reads, analysis of Sanger reads of a 40-kb insert library (strain JEL423), and comparison of chromosome copy numbers and loss of heterozygosity (LOH) (8–10). Contigs were only fused/extended if the RTP6 PacBio reads mapped uniquely to the newly fused contig. Following manual curation of the genome structure, Illumina paired-end (PE) reads from B. dendrobatidis strain RTP5 (Dunedin, New Zealand) were mapped to each contig. RTP5 appears to be identical to strain RTP6 based on single nucleotide polymorphisms (SNPs) and LOH patterns. Regions with <1.5× the basal coverage of each contig (i.e., nonrepetitive regions) were error corrected using the Illumina reads. The final assembly consisted of 63 contigs (59 nuclear, 4 mitochondrial) with a total length of 24.102 Mb (*N*_50_, 1,511 kb).

Telomeric repeat structures, identical to a canonical telomeric sequence (TTAGGG)_n_, were identified at the termini of 7 contigs (11). Immediately adjacent to each telomere was a region of 20 to 80 kb containing numerous highly repetitive sequences resembling subtelomeric domains. The highly repetitious sequences meant that it was impossible to accurately link these subtelomeric regions to other contigs.

The entirety of the nuclear genome in the assembly was phased using LOH regions of 56 global B. dendrobatidis strains. The genomes of B. dendrobatidis isolates commonly exhibit LOH as a consequence of mitotic recombination (8). This results in large chromosome regions becoming homozygous. Illumina reads for 56 strains of B. dendrobatidis were mapped to all contigs, and nonrepetitive variant frequencies were graphed against their positions in each contig. LOH regions were detected as areas of homozygosity where other strains were heterozygous, allowing the phasing of entire chromosome blocks. Although B. dendrobatidis is generally described as diploid, many isolates show trisomy for particular chromosomes. Trisomic regions display 2:1 SNP frequencies; this phenomenon was used to confirm and complete the phasing.

### Data availability.

This whole-genome shotgun project has been deposited at DDBJ/ENA/GenBank under the accession number QUAD00000000. The version described in this paper is version QUAD01000000. PacBio read data for B. dendrobatidis strain RTP6 and Illumina read data for the 10 B. dendrobatidis strains sequenced in this study were deposited in the NCBI SRA under BioProject accession number PRJNA483086.

## References

[1] Berger L, Speare R, Daszak P, Green DE, Cunningham AA, Goggin CL, Slocombe R, Ragan MA, Hyatt AD, McDonald KR, Hines HB 1998 Chytridiomycosis causes amphibian mortality associated with population declines in the rain forests of Australia and Central America. Proc Natl Acad Sci U S A 95:9031–9036. doi:10.1073/pnas.95.15.9031. 9671799 PMC21197

[2] Longcore JE, Pessier AP, Nichols DK 1999 *Batrachochytrium dendrobatidis* gen. et sp. nov., a chytrid pathogenic to amphibians. Mycologia 91:219–227. doi:10.2307/3761366.

[3] Lips KR 2016 Overview of chytrid emergence and impacts on amphibians. Philos Trans R Soc B 371:20150465. doi:10.1098/rstb.2015.0465. PMC5095542 28080989

[4] Farrer RA, Weinert LA, Bielby J, Garner TWJ, Balloux F, Clare F, Bosch J, Cunningham AA, Weldon C, Du Preez LH, Anderson L, Pond SLK, Shahar-Golan R, Henk DA, Fisher MC 2011 Multiple emergences of genetically diverse amphibian-infecting chytrids include a globalized hypervirulent recombinant lineage. Proc Natl Acad Sci U S A 108:18732–18736. doi:10.1073/pnas.1111915108. 22065772 PMC3219125

[5] Rhoads A, Au KF 2015 PacBio sequencing and its applications. Genomics Proteomics Bioinformatics 13:278–289. doi:10.1016/j.gpb.2015.08.002. 26542840 PMC4678779

[6] Longcore J 2000 Culture technics for amphibian chytrids: recognizing, isolating, and culturing Batrachochytrium dendrobatidis from amphibians, pp 52–54. *In* Proceedings of Getting the Jump! On Amphibian Diseases Conference/Workshop, Cairns, Australia.

[7] Koren S, Walenz BP, Berlin K, Miller JR, Bergman NH, Phillippy AM 2017 Canu: scalable and accurate long-read assembly via adaptive k-mer weighting and repeat separation. Genome Res 27:722–736. 28298431 10.1101/gr.215087.116 PMC5411767

[8] Symington LS, Rothstein R, Lisby M 2014 Mechanisms and regulation of mitotic recombination in *Saccharomyces cerevisiae*. Genetics 198:795–835. doi:10.1534/genetics.114.166140. 25381364 PMC4224172

[9] Farrer RA, Henk DA, Garner TW, Balloux F, Woodhams DC, Fisher MC 2013 Chromosomal copy number variation, selection and uneven rates of recombination reveal cryptic genome diversity linked to pathogenicity. PLoS Genet 9:e1003703. doi:10.1371/journal.pgen.1003703. 23966879 PMC3744429

[10] Sumpter NA 2018 Hybrid assembly of the chytrid genome: insights into the evolution and outbreak of a major amphibian pathogen. Masters dissertation. University of Otago, Dunedin, New Zealand.

[11] Zakian VA 1989 Structure and function of telomeres. Annu Rev Genet 23:579–604. doi:10.1146/annurev.ge.23.120189.003051. 2694944

[12] O’Hanlon SJ, Rieux A, Farrer RA, Rosa GM, Waldman B, Bataille A, Kosch TA, Murray KA, Brankovics B, Fumagalli M, Martin MD, Wales N, Alvarado-Rybak M, Bates KA, Berger L, Böll S, Brookes L, Clare F, Courtois EA, Cunningham AA, Doherty-Bone TM, Ghosh P, Gower DJ, Hintz WE, Höglund J, Jenkinson TS, Lin C-F, Laurila A, Loyau A, Martel A, Meurling S, Miaud C, Minting P, Pasmans F, Schmeller DS, Schmidt BR, Shelton JMG, Skerratt LF, Smith F, Soto-Azat C, Spagnoletti M, Tessa G, Toledo LF, Valenzuela-Sánchez A, Verster R, Vörös J, Webb RJ, Wierzbicki C, Wombwell E, Zamudio KR, Aanensen DM, James TY, Gilbert MTP, et al 2018 Recent Asian origin of chytrid fungi causing global amphibian declines. Science 360:621–627. doi:10.1126/science.aar1965. 29748278 PMC6311102

